# Human Fecal Contamination Corresponds to Changes in the Freshwater Bacterial Communities of a Large River Basin

**DOI:** 10.1128/Spectrum.01200-21

**Published:** 2021-09-08

**Authors:** Jill S. McClary-Gutierrez, Zac Driscoll, Cheryl Nenn, Ryan J. Newton

**Affiliations:** a School of Freshwater Sciences, University of Wisconsin–Milwaukee, Milwaukee, Wisconsin, USA; b Milwaukee Riverkeeper, Milwaukee, Wisconsin, USA; University of Minnesota

**Keywords:** bacterial community, fecal pollution, rivers, sewage

## Abstract

Microbial water quality is generally monitored by culturable fecal indicator bacteria (FIB), which are intended to signal human health risk due to fecal pollution. However, FIB have limited utility in most urbanized watersheds as they do not discriminate among fecal pollution sources, tend to make up a small fraction of the total microbial community, and do not inform on pollution impacts on the native ecosystem. To move beyond these limitations, we assessed entire bacterial communities and investigated how bacterial diversity relates to traditional ecological and human health-relevant water quality indicators throughout the Milwaukee River Basin. Samples were collected from 16 sites on 5 days during the summer, including both wet and dry weather events, and were processed by 16S rRNA gene amplicon sequencing. Historical water quality at each sampling location, as opposed to upstream land use, was associated significantly with bacterial community alpha diversity. Source partitioning the sequence data was important for determining water quality relationships. Sewage-associated bacterial sequences were detected in all samples, and the relative abundance of sewage sequences was strongly associated with the human *Bacteroides* fecal marker. From this relationship, we developed a preliminary threshold for human sewage pollution when using bacterial community sequence data. Certain abundant freshwater bacterial sequences were also associated with human fecal pollution, suggesting their possible utility in water quality monitoring. This study sheds light on how bacterial community analysis can be used to supplement current water quality monitoring techniques to better understand interactions between ecological water quality and human health indicators.

**IMPORTANCE** Surface waters in highly developed mixed-use watersheds are frequently impacted by a wide variety of pollutants, leading to a range of impairments that must be monitored and remediated. With advancing technologies, microbial community sequencing may soon become a feasible method for routine evaluation of the ecological quality and human health risk of a water body. In this study, we partnered with a local citizen science organization to evaluate the utility of microbial community sequencing for identifying pollution sources and ecological impairments in a large mixed-use watershed. We show that changes in microbial community diversity and composition are indicative of both long-term ecological impairments and short-term fecal pollution impacts. By source partitioning the sequence data, we also estimate a threshold target for human sewage pollution, which may be useful as a starting point for future development of sequencing-based water quality monitoring techniques.

## INTRODUCTION

Mixed-use watersheds can be impaired by pollutants from many different sources, such as urban runoff, sewage overflows, agricultural runoff, livestock, or wildlife. These sources can contribute a range of pollutants, including pathogens, sediment, or nutrients, which can lead to deteriorating ecological water quality and/or increased human health risk. Current microbial water quality monitoring methods based on detection of culturable fecal indicator bacteria (FIB) can produce highly variable results, suggesting that evaluating a single indicator may not be appropriate for determining health risk (1, 2). Identifying the causes of specific pollution events or persistent water quality impairments is an important step in initiating new watershed-scale protection efforts (3–6).

Microbial source tracking (MST) is one technique used to identify the sources of fecal pollution in a water body. MST typically uses quantitative PCR methods to detect 16S rRNA gene fragments from bacteria that are sufficiently specific to an environmental source of interest (7–9). The majority of MST assays for fecal pollution tracking are designed to identify bacteria specific to the gastrointestinal tracts of certain animals (such as dogs or ruminants) (10, 11) or humans (12), though additional MST assays for identifying sewage as opposed to human feces are also being developed (13). MST assays have been successfully applied to identify pollution sources in various locations (14–19). Despite their demonstrated utility, MST requires sufficient prior knowledge of the pollution source of interest in order to design an appropriately specific assay (20–22). Additionally, it is highly likely that no MST assay will ever demonstrate perfect specificity with its intended source, leading many to recommend the use of multiple MST assays (21).

As DNA sequencing can identify many potential microbes of concern in a single sample, it may prove advantageous over single MST assays for profiling risks and impairments in a broad range of water environments. Most previous applications of DNA sequencing in water environments have focused on broadly describing the impact of pollutants or processes on bulk microbial communities in particular sample types (23–27). However, as water environments are often highly complex, with a mix of active or inactive community members and contributions from various natural or anthropogenic sources, observations of bulk microbial community composition may not be appropriate for identifying pollutant monitoring targets, especially when pollutants may be present at low levels. Methods to utilize sequencing data for pollution tracking, such as SourceTracker (28) and FORENSIC (29, 30), are being developed. While these approaches are useful, they are limited by a need for input of both pollutant and environmental community data (SourceTracker) or limited fecal pollution targets (FORENSIC), and neither method has been connected to health risk. With the plethora of sequencing data from a wide range of sample types and environments now available in public repositories, additional improvements can be made to identify pollutant thresholds related to human health or environmental health risks. Furthermore, coupling assessment of ecological and environmental conditions with specific pollution indicators could lead to the identification of new indicators or conditions that promote pollutant persistence (12). While DNA sequencing is not yet feasible for routine monitoring, new technologies that reduce both cost and time to generate results are actively being developed and validated (31, 32). In parallel with developments in technology, we need further developments in data analysis if we are to capitalize on the advantages that sequencing microbial communities provide for source tracking.

The goal of this research is to evaluate the use of microbial community diversity and composition as parameters for evaluating river and stream water quality. By assessing changes in microbial community diversity and partitioning communities against curated databases, we provide insights into how DNA sequencing data can be incorporated into monitoring frameworks for both ecological and human health.

## RESULTS

### Location description.

**FIG 1:**
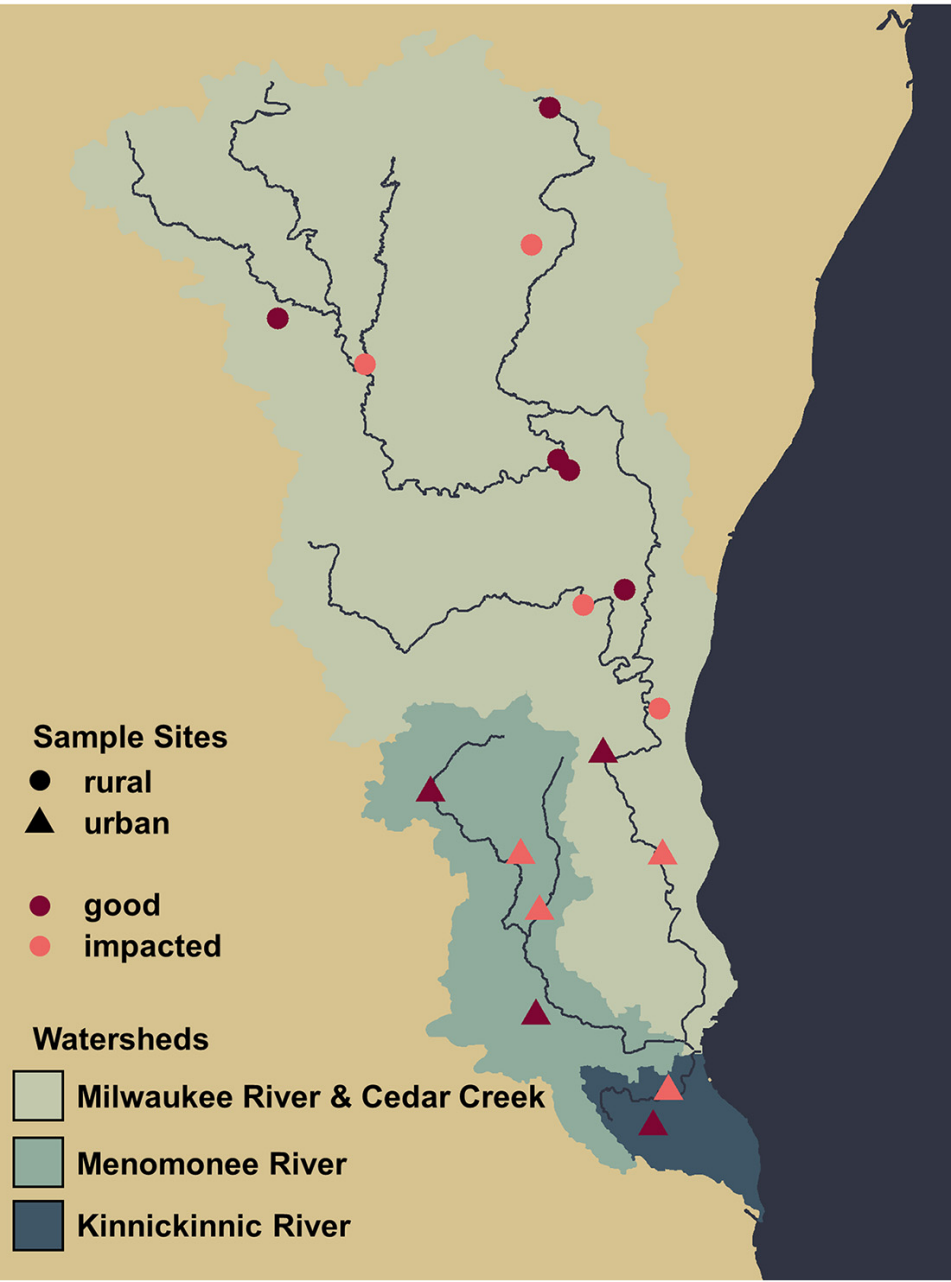
Map of sampling sites and watersheds. Sampling sites are designated urban or rural and good or impacted water quality.

The Milwaukee River watershed is a large mixed-land-use watershed containing a total of 875 miles of stream reaches that divide into three main rivers, the Milwaukee, Menomonee, and Kinnickinnic Rivers, which flow into the Milwaukee harbor estuary and Lake Michigan. Sampling sites included different stretches of the rivers themselves but mostly consisted of smaller tributary streams. Land uses range from largely agricultural in the north to densely urban in the south, and 46% of stream reaches within the basin are considered impaired by at least one pollutant (33). Overall, sampling sites represented first- through fourth-order streams, with upstream watershed areas ranging from 1.8 to 117 square miles. Upstream watersheds of each sampling site also ranged in land use from 3% to 100% developed land based on U.S. Geological Survey (USGS) data. In general, sites were considered “urban” in the south and “rural” in the north of the Milwaukee River Basin. In the individual sites’ upstream watersheds, rural sites contained 3 to 23% developed land and urban sites contained 24 to 100% developed land (see Table S1 in the supplemental material). Samples were collected from 16 different sites in the Milwaukee River watershed on five separate days between June and August 2017 (Fig. 1).

### Bacterial community diversity is higher in historically high-quality streams and correlates with physicochemical measurements.

**FIG 2:**
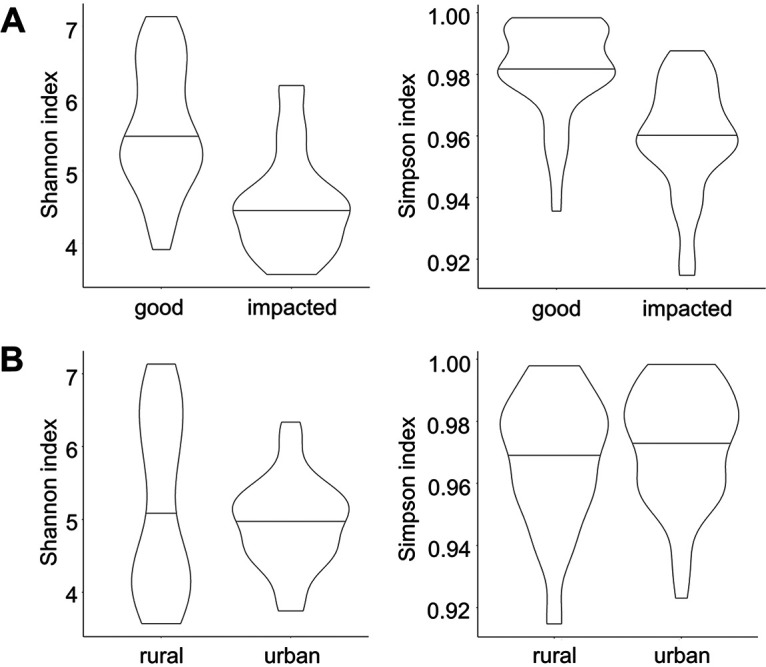
Alpha diversities (calculated using Shannon and Simpson indices) of microbial communities at good versus impacted sites (A) and urban versus rural sites (B).

In total, we detected more than 10,797 unique amplicon sequence variants (ASVs) in our DNA sequencing data. Each individual sample contained no more than a maximum of 2,402 ASVs, and 268 ASVs were only detected at one sampling site. Across all samples, only 1,488 ASVs were detected in more than half of sampling sites, demonstrating the diversity of bacterial communities from site to site and the complexity in determining indicators of water quality and human health risks throughout the watershed. We found a statistically significant decrease in bacterial community diversity in impacted streams compared to unimpacted streams using two different metrics for diversity (Wilcoxon rank sum test, *P* < 0.01) (Fig. 2A). In contrast, there was no significant difference in alpha diversity metrics for urban versus rural sampling sites (Fig. 2B), but we did observe a statistically significant correlation between developed land area and Shannon diversity across the sites sampled (Spearman rank sum test, ρ = 0.50, *P* < 0.05) (Fig. S1 in the supplemental material), with higher Shannon diversity associated with a lower proportion of developed land area in the upstream watershed.

**FIG 3:**
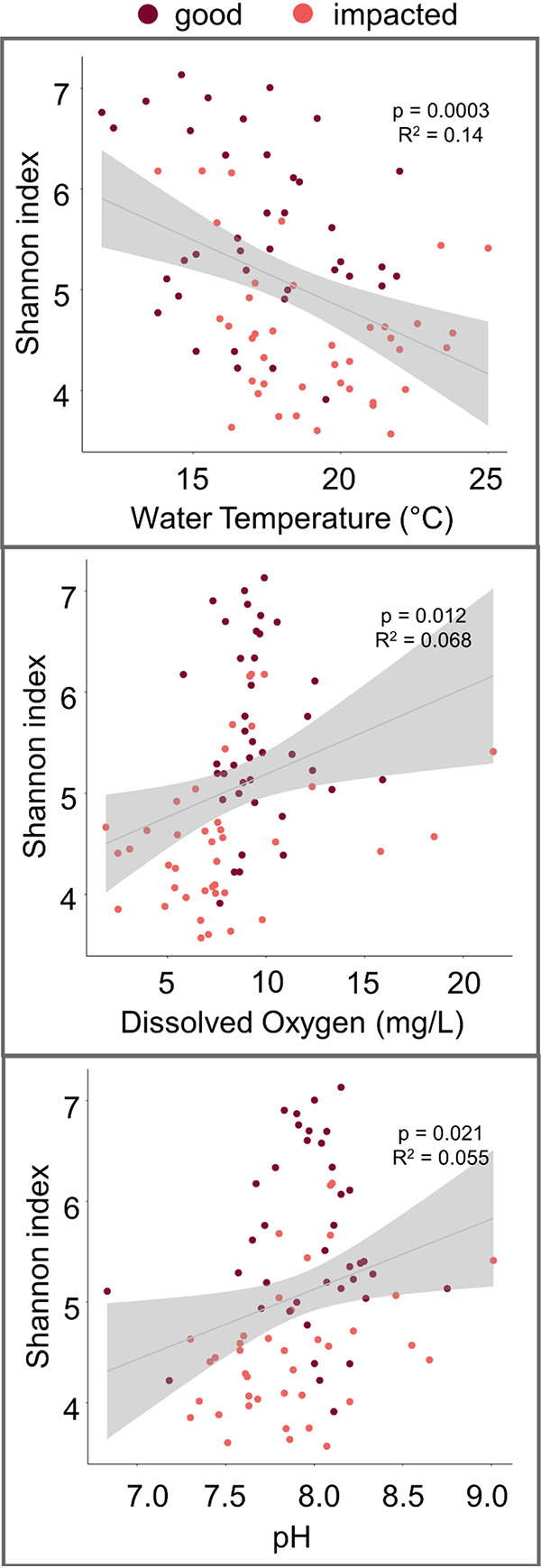
Correlation between alpha diversity (Shannon index) and water temperature, dissolved oxygen, and pH. Shaded regions are 95% confidence intervals around linear model fits.

**FIG 4:**
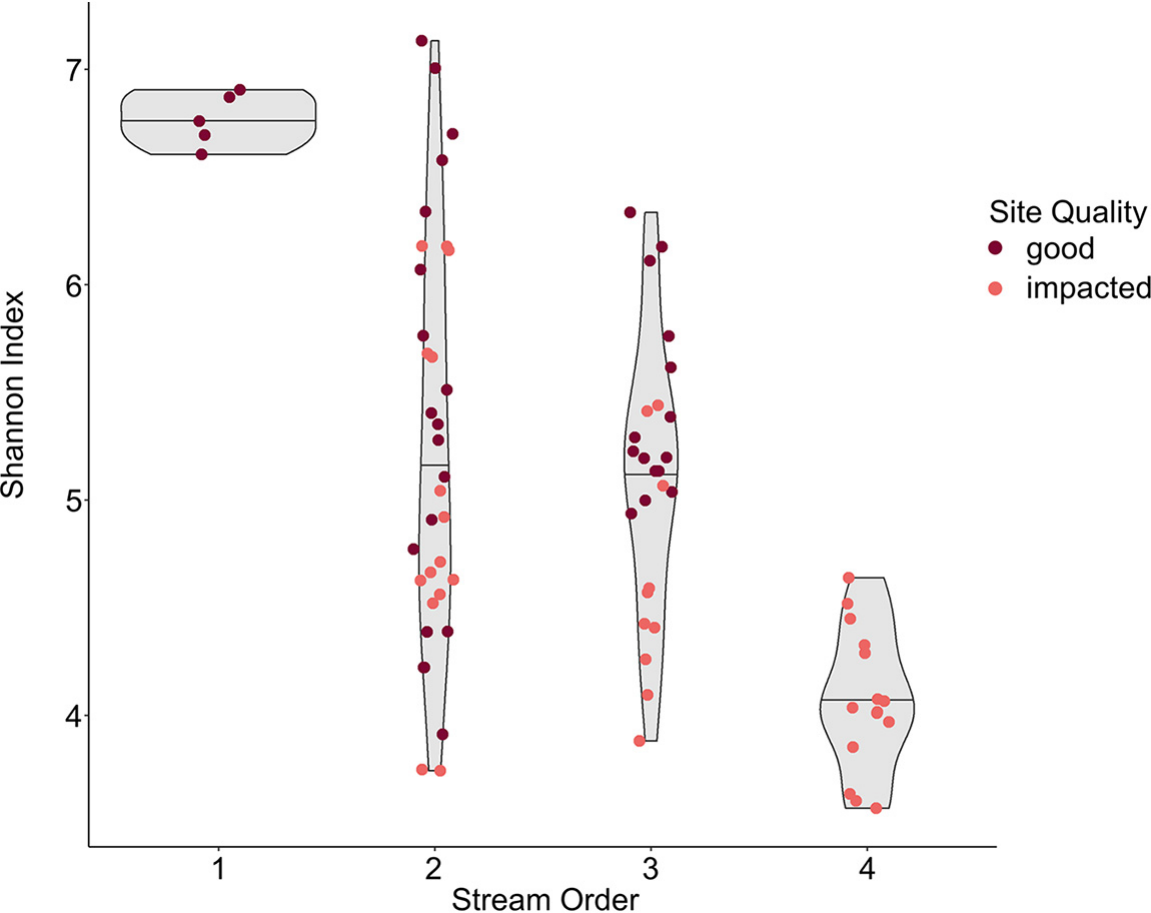
Relationship between stream order and alpha diversity (Shannon index). Points represent individual samples, and the widths of gray shaded violins represent the distributions of the individual samples. Black horizontal lines are medians of point distributions.

Bacterial community alpha diversity was significantly correlated with temperature, dissolved oxygen (DO), and pH (Fig. 3), suggesting that streams in this region with lower temperature, higher DO, and higher pH tended to be characterized by more diverse microbial communities. However, correlations between these instantaneous water quality measurements and bacterial community diversity were generally weak, suggesting that these variables do not explain much of the variability in community diversity between sites. DO and pH were also correlated with each other, suggesting that possibly only one of these variables influences bacterial community diversity. In addition, we found that bacterial community alpha diversity was significantly correlated with stream order, with higher diversity in lower-order streams (Fig. 4).

### Sewage sequences are detected in all samples.

**FIG 5:**
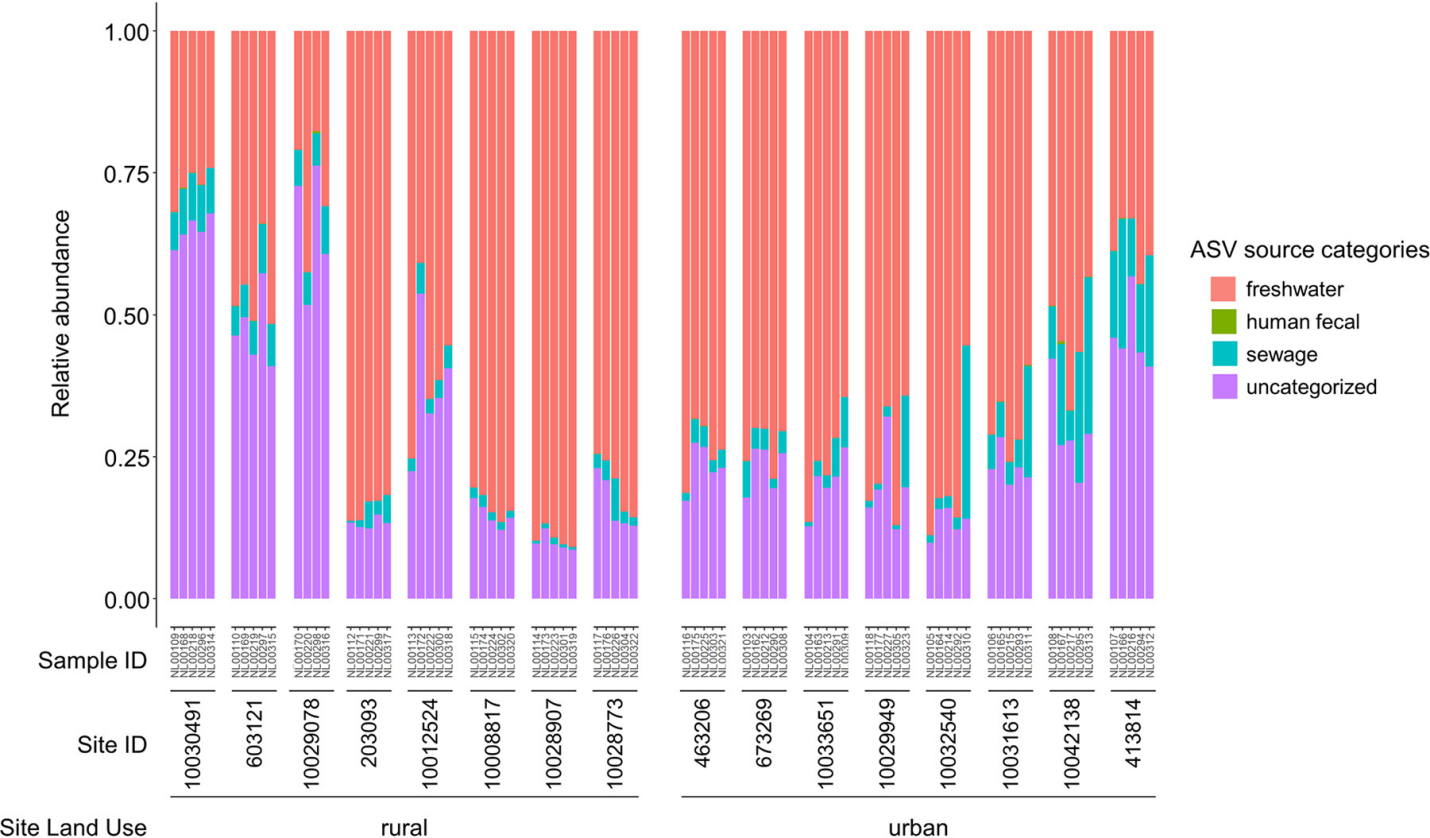
Relative abundances of ASVs across freshwater, human fecal, and sewage source categories. Uncategorized sequences are those that could not be assigned to any of the other categories. Each bar represents a single sample. Sites within rural and urban categories are organized by site location latitude (i.e., north to south).

**FIG 6:**
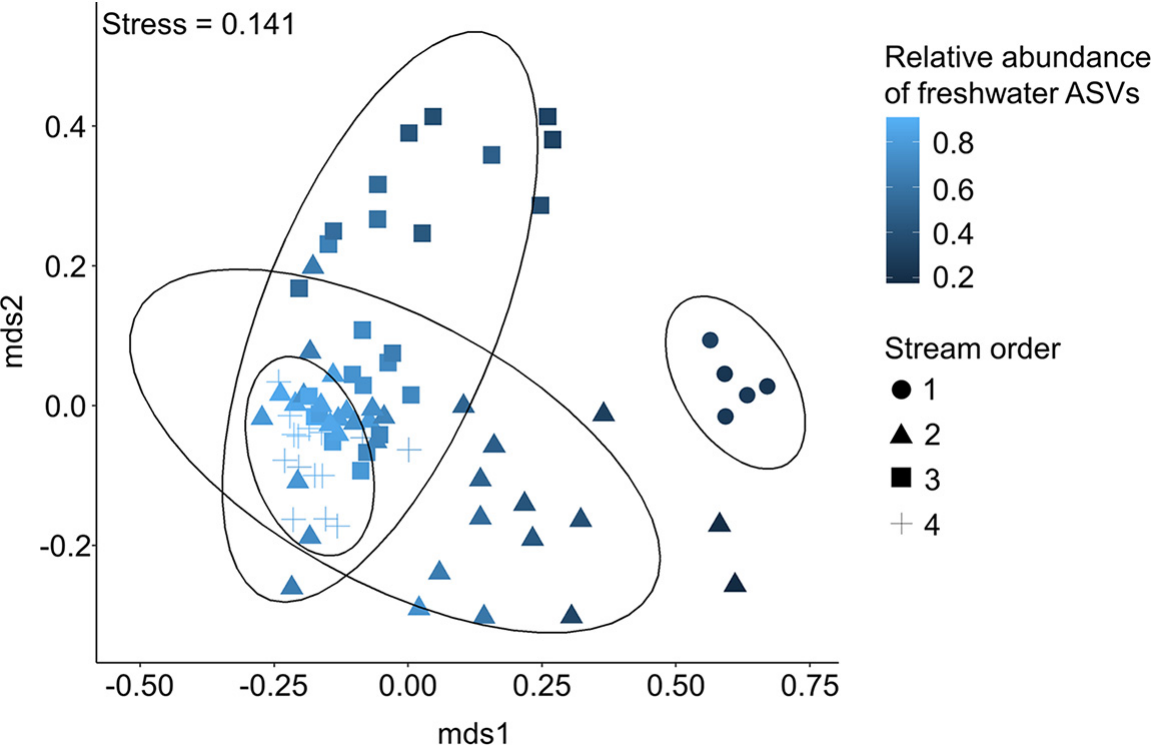
Nonmetric multidimensional scaling of microbial communities based on Bray-Curtis dissimilarity. Data ellipses designate groupings by stream order.

**FIG 7:**
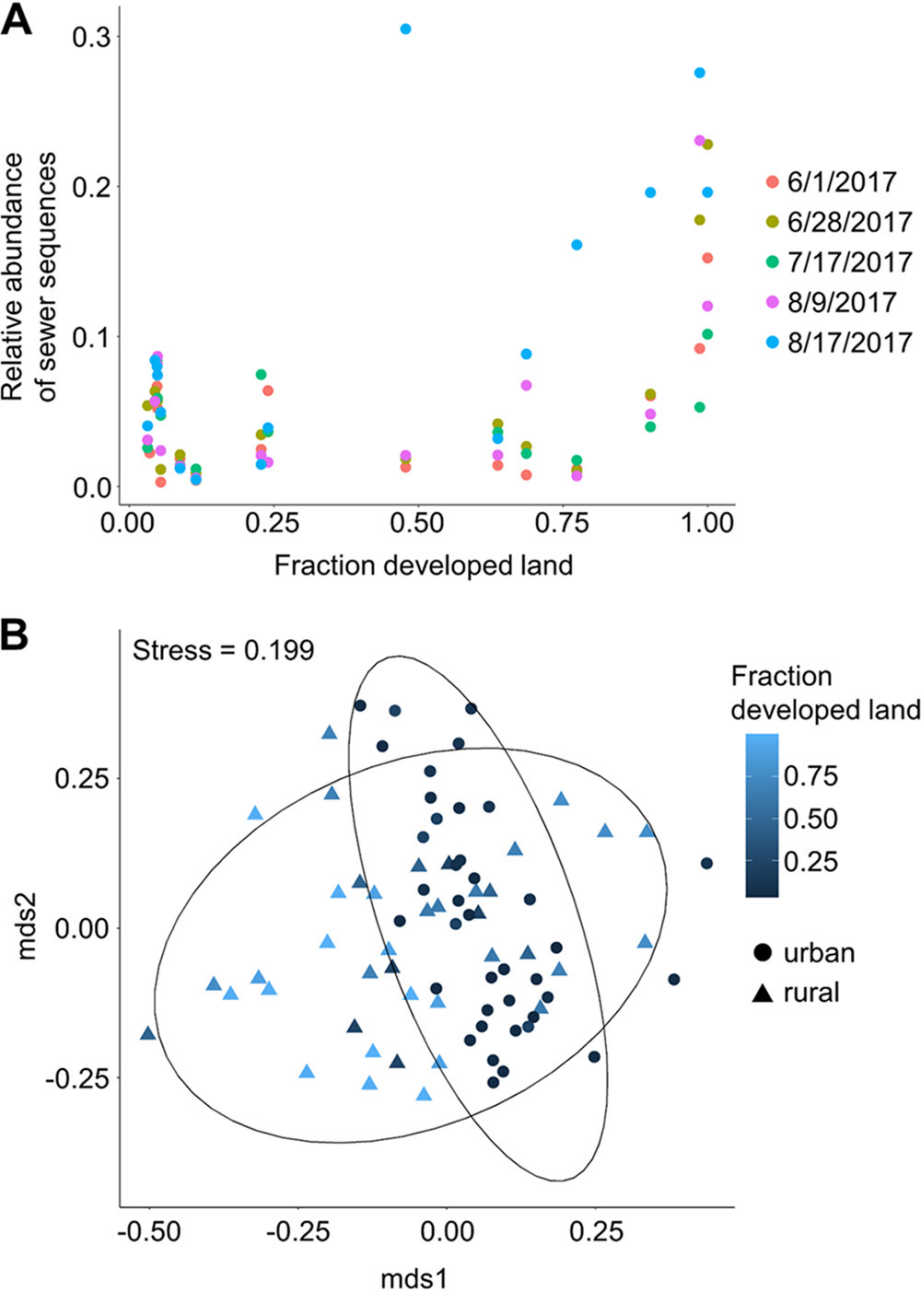
(A) Relationship between proportion of sewage sequences and developed land. (B) Relationship between composition of sewage sequences and land use. Nonmetric multidimensional scaling is based on Bray-Curtis dissimilarity of sewage sequences only. Data ellipses designate groups based on dominant land use.

Overall, the percentages of sequences attributed to freshwater or sewage sources varied widely across samples (Fig. 5). Freshwater sequences made up 15% to 90% of total sequences in each sample collected. By visualizing Bray-Curtis dissimilarity using a nonmetric multidimensional scaling (NMDS) ordination, samples with a high percentage of freshwater sequences clustered together and were made up of mostly fourth-order streams (Fig. 6). Sewage sequences were detected in all samples collected, making up between 0.3% and 30% of total sequences in each sample. Within the sewage sequences, we identified those most likely originating from human feces or of sewer origin (sewer associated). Across all water samples, a total of 361 unique ASVs were identified as probable sewer sequences, and an additional 16 ASVs were identified as probable human fecal sequences. Human fecal sequences were detected in 37 of 79 samples, making up between 0.003% and 0.5% of total sequences in samples where they were detected. The proportion of sewage sequences in each sample generally increased with increasing developed land proportions in the watershed, though this relationship was not significant (Spearman rank sum test, *P* = 0.10) (Fig. 7A). The proportion of sewage sequences appeared to also be influenced by sampling date; nearly half (7/16) of the sites exhibited their highest proportions of sewage sequences on August 17, which was the only sampling date associated with wet weather (Fig. 7A). By considering the 377 total human fecal and sewer-derived ASVs, we found that the beta diversity of this community was significantly associated with both sample site latitude and urban versus rural land use (PERMANOVA, adonis function [34], *P < *0.01) (Fig. 7B), suggesting significant spatial variation in human sewage sequence distribution throughout the basin. Human sewage community composition was also significantly associated with both water temperature and DO (PERMANOVA, *P < *0.01), but not pH (*P = *0.12). However, correlation coefficients were again quite low, indicating weak explanatory power of these variables.

### Connection between sequencing-based human sewage and freshwater microbial communities and HB qPCR abundance.

Using quantitative PCR (qPCR), human *Bacteroides* (HB) was detected in 66% of all samples collected, and the concentrations in these samples ranged from below the limit of quantification (<180 copy number [CN]/100 ml) to 26,442 CN/100 ml. HB concentrations were significantly associated with the compositions of both human sewage and freshwater microbial communities (permutational multivariate analysis of variance [PERMANOVA], *P* < 0.001). Additionally, while no association was observed between quantifiable HB concentrations and the proportions of ASVs assigned to freshwater bacteria (Spearman rank sum test, *P* = 0.2), a significant rank correlation was observed between HB concentrations and the proportions of sewer and fecal sequences (*P* = 0.02). This relationship between HB concentrations, which are already used in health risk assessments, and sewer-associated sequences suggests that the relative abundance of sewer-associated organisms detected by sequencing may be a useful indicator of human health risk as sequencing technologies develop and become more appropriate for routine use.

**FIG 8:**
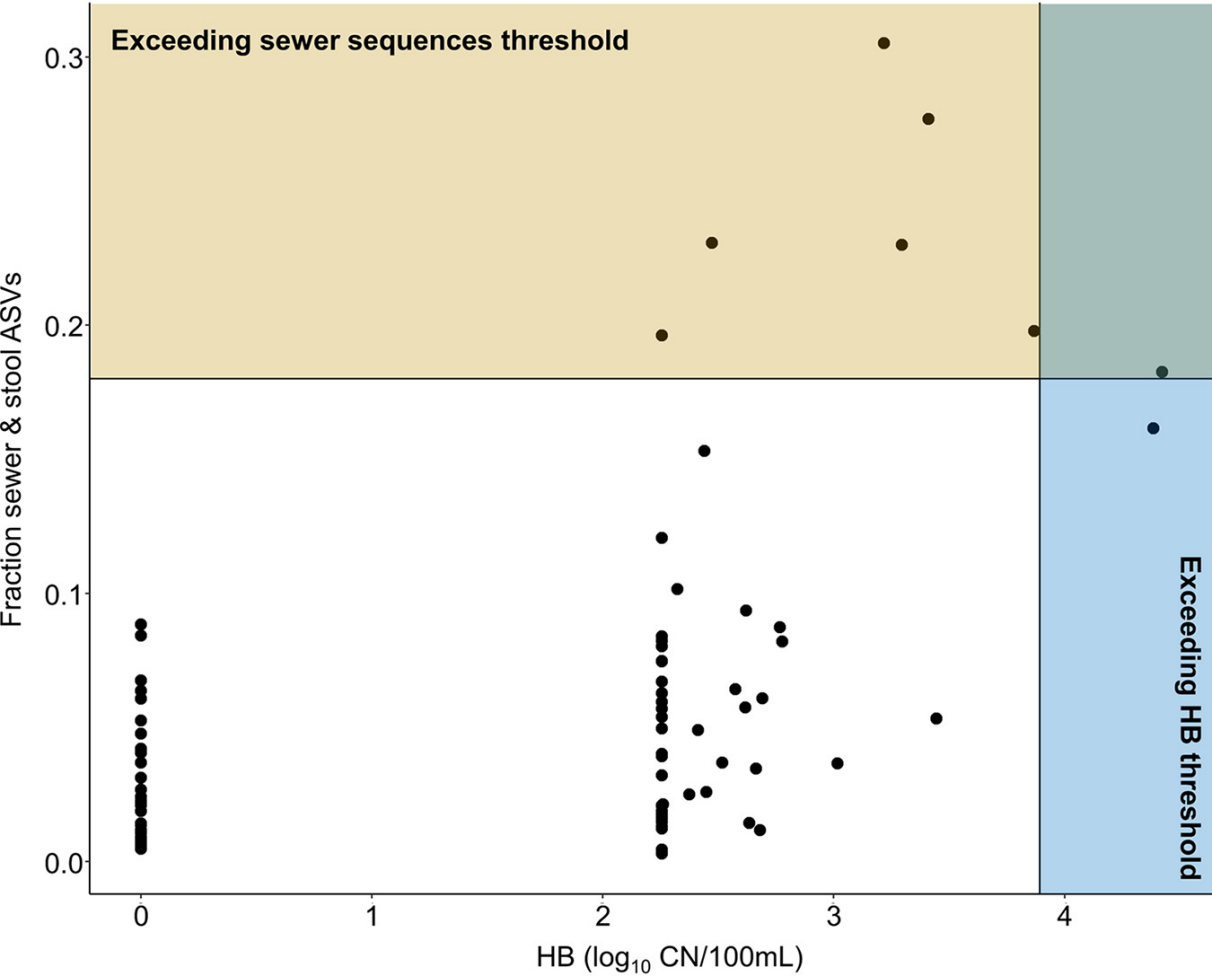
Comparison of sewer sequence relative abundances and human *Bacteroides* (HB) concentrations across all samples. Samples exceeding the HB concentration threshold appear in the blue shaded region; samples exceeding the sewer sequence threshold appear in the yellow shaded region.

To explore this possibility, we used a Monte Carlo simulation to convert a previously defined risk-based threshold of HB (7,800 CN/100 ml [16]) into an expected corresponding threshold of sewage sequence relative abundance (see supplemental materials for further details). From this simulation, we estimate that a threshold of 18% sewage sequences corresponds to significant human sewage pollution. A comparison of these two thresholds for the samples collected in this study is shown in Fig. 8. Seventy-two of 79 samples demonstrate agreement between the two thresholds, with 71 samples appearing below both thresholds and one sample above both thresholds. Six of 79 samples exceed the sewer sequence threshold only, and one sample exceeds the HB threshold only. Error on our sequence proportion estimate remains relatively large (±1 geometric standard deviation = 2% to 200%) and is primarily due to high variability in the distribution of total cell concentrations in rivers (see the supplemental material).

To determine specifically which, if any, ASVs in these communities were indicative of samples with elevated HB concentrations, we categorized samples based on their HB concentrations in categories of undetectable (below limit of detection), unquantifiable (detected but below limit of quantification), quantifiable (180 to 7,800 CN/100 ml), and risk (≥7,800 CN/100 ml). Since land use was determined previously to impact the microbial community composition, we performed this analysis only on samples from urban catchment areas. Among sewer and fecal ASVs, four ASVs (assigned to Bacteroides dorei, Bacteroides uniformis, *Trichococcus* sp., and *Leuconostoc* sp.) were identified as indicative of HB contamination above the risk threshold, one ASV was indicative of detectable HB contamination, one ASV of HB contamination below the risk threshold, and one ASV of unquantifiable (Table 1). Of the seven total sewer and stool ASVs identified, only the two *Bacteroides* ASVs associated with HB contamination above the risk threshold were from human stool communities (as opposed to sewer pipe communities). Among freshwater ASVs, 4 ASVs were associated with HB contamination above the risk threshold, 2 ASVs were associated with quantifiable HB contamination, 11 with detectable HB contamination, 10 with HB contamination below the risk threshold, and 3 with unquantifiable HB contamination (Table 1). Of the 30 total freshwater ASVs identified by indicspecies, 8 were assigned to the genus *Flavobacterium*.

**TABLE 1:** ASVs strongly associated with different human *Bacteroides* contamination categories in urban samples^*a*^

HB category	Sewer and stool community sequences				Freshwater community sequences			
	ASV ID^*b*^	Taxonomy	Specificity	Sensitivity	ASV ID	Taxonomy	Specificity	Sensitivity
Risk (≥7,800 CN/100 ml)	5850	Bacteroides dorei	1	1	1802	*Flavobacterium* sp.	0.9611	1
	5624	Bacteroides uniformis	0.9276	1	3070	*Novosphingobium* sp.	0.8907	1
	955	*Trichococcus* sp.	0.8995	1	4335	Unclassified *Enterobacteriaceae*	0.8515	1
	681	*Leuconostoc* sp.	0.8229	1	508	Enterobacter sp.	0.8309	1

Quantifiable (>LOQ)					342	*Flavobacterium* sp.	0.9333	0.8824
					585	Flavobacterium chungangense	0.8852	0.8235

Detectable (>LOD)	116	Hydrogenophaga taeniospiralis	0.9483	1	35	*Flavobacterium* sp.	0.9901	1
					258	*Flavobacterium* sp.	0.9572	1
					298	Flavobacterium segetis	0.9448	0.9643
					341	Gemmobacter aquatilis	0.9494	0.9286
					103	*Flavobacterium* sp.	0.9228	0.9286
					96	*Flavobacterium* sp.	0.9892	0.8571
					248	*Pseudorhodobacter* sp.	0.9703	0.8571
					203	*Sediminibacterium* sp.	0.9667	0.8571
					155	*Rhodoferax* sp.	0.9515	0.8571
					142	Acinetobacter sp.	0.9845	0.8214
					462	*Lelliottia* sp.	0.9717	0.8214

No risk (<7,800 CN/100 ml)	333	*Reyranella* sp.	1	0.9211	21	*Fluviicola* sp.	1	0.9211
					28	*Rhodoluna* sp.	0.9793	0.9211
					52	“*Candidatus* Planktophila sp.”	1	0.8947
					58	“*Candidatus* Planktoluna sp.”	1	0.8947
					139	MWH-Ta3 sp.	11	0.8684
					160	*Tabrizicola* sp.		0.8684
					204	*Sediminibacterium* sp.	1	0.8684
					22	Unclassified *Burkholderiaceae*	1	0.8684
					233	Unclassified *Sporichthyaceae*	1	0.8158
					344	hgcl_clade sp.	1	0.8158

Unquantifiable (<LOQ)	351	Unclassified *Steroidobacteraceae*	0.8659	0.8696	55	“*Candidatus* Limnoluna sp.”	0.8599	0.8696
					144	*Sediminibacterium* sp.	0.8776	0.8261
					187	“*Candidatus* Planktoluna sp.”	0.8214	0.8696

a Strongly associated ASVs were those with specificity and sensitivity both ≥80%.

b ID, identification number.

## DISCUSSION

Large mixed-use watersheds like the Milwaukee River Basin can be affected by many different pollutants, leading to a range of impairments and problems that must be addressed by water quality monitoring efforts. Methods that allow combined assessment of ecological quality and human health risk would be valuable to streamline both monitoring and mitigation. Bacterial community DNA sequencing is a rapidly advancing technique that can provide information on the overall quality of a watershed and simultaneously detect specific microbes that may be associated with transient or continuous human health-relevant pollution sources. While using DNA sequencing for routine water quality monitoring remains generally prohibitive due to the cost and expertise required, sequencing methods are quickly advancing to address these difficulties. Our goal in this project was to apply DNA sequencing in conjunction with typical water quality monitoring performed by a local citizen science organization to determine its potential future utility in describing the overall quality of the watershed and identifying possible pollution sources to address.

Dissolved oxygen (DO) has been shown to significantly influence microbial community composition in various water bodies (35, 36). In particular, a previous study identified positive correlations between DO and bacterial community alpha diversity in a Great Lakes watershed (37), and our study corroborates these relationships in the Milwaukee River Basin. As the site-specific upstream watersheds varied widely in size in our study, we hypothesized that bacterial community diversity might be impacted more strongly by the overall size of the developed area as opposed to the percentage of developed area in the upstream watershed. In our system, as streams travel from first to fourth order, changes in water chemistry, including increased temperature and decreased pH, appear to also decrease bacterial community diversity. While others have observed positive associations between alpha diversity and temperature or flow rate (38, 39), in this watershed, as communities travel from headwaters to Lake Michigan, they are likely to develop into communities more closely resembling typical freshwater lake communities, which tend to have lower diversity than terrestrial sources (40). Previous studies have also shown declines in bacterial diversity in river systems from upstream to downstream (41, 42), though Jordaan and Bezuidenhout observed the opposite in a highly urbanized river (43). Thus, the relationship between bacterial community diversity and land use may be less a factor of land use impacts on the water body and more related to maturation and development of the freshwater microbial community. This highlights the difficulty in establishing a threshold for alpha diversity to assess water quality, as the “ideal” alpha diversity will vary with other factors, such as stream order, trophic status, or possibly land use. However, the strong correlation observed in our data between alpha diversity and historical water quality suggests promise for this method, and future research should focus on understanding how alpha diversity varies within stream order classifications and across land use and water quality environmental variables.

While the HB marker is considered highly specific for the discrimination of human fecal inputs from other fecal pollutants for microbial source tracking, its widespread utility for risk assessment is hampered by inconsistencies in its presence and stability across global populations (21, 44–46), along with variable decay or transport between the HB marker and human pathogens (47–49). In contrast to this single-marker approach, which is prone to more random source input variation, our sewage community proportion threshold represents a full community approach that essentially incorporates hundreds of individual indicators to create a more robust and sensitive signal of sewage pollution. While detection of DNA via qPCR or sequencing does not discriminate between live and dead organisms, sewage exposure is strongly linked to health risk in epidemiological studies (50–52). Therefore, identification of high proportions of the full sewage community may serve as a more conservative indicator of health risk than qPCR markers and could be applied as a useful benchmark for the development of sequencing-based water quality assessments. Here, we have used a mass balance and Monte Carlo simulation approach to estimate a threshold based on sewage sequence abundance. However, we note that our estimation is relatively simple and should be further refined by considering other factors. For example, as our proportion estimate is based on total cell concentrations in river water being relatively stable, a high influx of another biological contaminant, such as animal feces, at the time of sampling could confound our sewage sequence proportion by contributing related fecal sequences and increasing the background total cell concentration. Additional data collection directed at understanding the effects of other variables (e.g., total bacterial cell concentrations, sequencing biases, flow rates, community composition shifts, etc.) on our sewage community proportion metric are needed to reduce error in our estimations and set more definitive thresholds.

In examining the human sewage community specifically, we observed that two human stool ASVs assigned to B. dorei and B. uniformis were indicative of high HB contamination. These two ASVs were only detected in three and five samples, respectively, and the combination of both ASVs was only detected in the two samples with high HB contamination above the health risk threshold. As the HB qPCR marker is designed for detection of a particular B. dorei strain (9, 11, 12), its association with two *Bacteroides* sequences suggests agreement between sequencing and qPCR methods. Furthermore, the lack of identification of any other human stool bacteria in high-HB samples suggests the possibility of differential decay between these markers and other sewer or human fecal bacteria. Additional sewage bacteria, including *Trichococcus* and *Leuconostoc* spp., were indicative of HB contamination above the risk threshold. *Trichococcus* spp. are typically present at high abundance in sewage (6, 53, 54).

Overall, the freshwater ASVs identified as associated with quantifiable HB levels were more abundant than human sewage sequences in the full community. In particular, 8 of 17 freshwater ASVs associated with HB detection were identified as *Flavobacterium* spp. Flavobacteria are commonly associated with urban environments and sewage-polluted waters (40), presumably because many members of this genus show strong growth responses in aquatic environments containing numerous complex carbon compounds (55). In addition to *Flavobacterium*, other freshwater bacteria typically associated with stormwater, wastewater infrastructure, and urban runoff, including members of Enterobacter, *Sediminibacterium*, Acinetobacter, and *Enterobacteriaceae* (6, 40, 56, 57), were also associated with HB detection. These freshwater ASVs are not expected to associate with health risk in all cases, as their detection may also be due to other organic matter inputs that do not comprise a human health risk. However, as real-time flow-based sequencing technologies detect higher-abundance sequences faster than rare sequences, these community members may be useful targets for the development of sequencing-based monitoring protocols for general contamination issues that warrant further evaluation. Finally, sequences identified as common freshwater organisms, including “*Candidatus* Planktophila,” “*Candidatus* Planktoluna,” and *Fluviicola* (40, 58, 59), were associated with no risk or unquantifiable HB. As these sequences are much more abundant in the water column than the *Bacteroides* sequences associated with high-risk HB concentrations, they may be useful for the development of markers of freshwater bodies with no or low impact.

### Conclusions.

Large mixed-use watersheds can be affected by many different pollution sources, and identifying the causes and effects of these pollutants remains a challenge. Despite their diversity of sizes, land uses, and flow paths, streams in large river basins are often regulated by basin-wide standards for pollutant loads and downstream impacts. In this study, we evaluated bacterial communities across a wide array of streams in a watershed with many water quality impairments, and we showed that higher bacterial community diversity is associated with historically healthy stream reaches. Furthermore, by evaluating subpopulations of bacterial communities associated with curated freshwater and sewage databases, we were able to more easily identify anthropogenic inputs at specific sites and potential impacts of these inputs on the typical freshwater community. While further technological advancements are needed before bacterial community analysis can be incorporated into routine monitoring, we demonstrate that this approach can provide useful information on both ecological and human health-relevant water quality metrics and that evaluation of disturbances in expected naturally occurring bacterial communities can provide insight into the prevalence of low-level sewage contamination.

## MATERIALS AND METHODS

### Sample collection.

Sample sites were selected to represent both southern (urban) and northern (rural) areas of the watershed, as well as streams with historically impacted or unimpacted quality. Sites were designated as impacted based on atypical dissolved oxygen (DO) concentrations in a long-term-monitoring data set (see the supplemental material) (60). Due to accessibility issues, only 15 sites were visited on the first sampling date (1 June 2017), and one site (number 10012525) was removed and replaced with site number 10012524 after the first sampling date. A total of 79 samples were collected. One sample date (17 August 2017) was during a wet weather event; all other samples were collected during dry weather periods. At the time of sample collection, physicochemical parameters (air temperature, water temperature, DO, pH, transparency, specific conductivity, and total phosphorus) were also measured using Wisconsin Department of Natural Resources recommended protocols for water quality monitoring (61). For each sample, surface water was collected into a 2-liter bottle and stored on ice for transport to the laboratory. Samples were processed immediately upon arrival at the laboratory (within 6 h of collection).

### Sample processing.

Two hundred fifty milliliters of each sample was vacuum filtered onto a 47-mm-diameter, 0.22-μm-pore-size mixed-cellulose ester filter (Millipore, Billerica, MA). Each filter was aseptically folded, inserted into a 2-ml screw-cap tube, and stored at −80°C until DNA extraction. DNA was extracted using a previously published protocol (62). In brief, samples were removed from the freezer and filters were crushed immediately using a sterile spatula. Crushed filter pieces were then added to FastDNA spin kit for soil bead-beating matrix tubes (MP Biomedicals, Solon, OH, USA). DNA extraction then proceeded following the manufacturer’s recommendations. After extraction, samples were stored at −20°C until sequencing.

### Library preparation and DNA sequencing.

Each DNA extract was amplified by targeting the V4-V5 hypervariable region of the 16S rRNA gene in triplicate using previously published PCR primers (518F and 926R) and conditions (63). A positive-control mock community (HM-783D, final error rate = 0.02% of sequenced nucleotides; BEI Resources, Manassas, VA) and a no-template control were also included in PCRs and carried through to sequencing. After PCR, triplicate amplicons from each sample were pooled and cleaned using the Agencourt AMPure XP kit (Beckman Coulter, Indianapolis, IN). Samples were then submitted to the Great Lakes Genomics Center, where they were barcoded, pooled, and sequenced on an Illumina MiSeq machine, generating 250-bp paired-end reads.

### Sequencing data processing.

Raw sequencing data were demultiplexed and quality scored by Illumina MiSeq software to generate fastq files for forward and reverse reads for each sample. PCR primers were first trimmed using cutadapt 1.18 from both forward and reverse reads in paired-end mode (64). Next, dada2 version 1.8.0 was used to filter input sequences by quality, generate amplicon sequence variants (ASVs), and remove chimeras (65). Additional quality filtering was performed in mothur version 1.41.1 (66) to screen out ASVs that were more than 5% shorter or longer than the median amplicon sequence length or were identical. Taxonomic assignments were then determined for ASVs using the SILVA version 132 taxonomy database and the assignTaxonomy and addSpecies functions in dada2. Following taxonomy assignment, ASVs that assigned to Eukarya, Chloroplast, or Mitochondria were removed, and any ASV present in fewer than three samples was also removed. Finally, the remaining ASVs were compared to the positive-control mock community ASVs. Of these, four mock community ASVs were identified in the samples, suggesting possible well-to-well contamination. These four ASVs were also removed from the sample data set.

To generate additional taxonomic assignments for typical freshwater organisms, TaxAss was used to assign ASVs to the FreshTrain database (58, 67). For human fecal and sewer organisms, a custom database was used based on a combination of DNA sequencing data from the Human Microbiome Project (68) and a large data set of raw sewage samples from Milwaukee (see the supplemental material) (69). Assignment to the human fecal and sewer ASV database was performed using a custom script to identify exact alignments (as in reference 69) (see the supplemental material), and any ASVs that were previously assigned to the freshwater database were excluded. River sequences that aligned exactly to human fecal or sewer sequences in the custom database were considered probable human fecal or sewer sequences if the 5th-percentile relative abundance in river samples was lower than the 95th-percentile relative abundance in raw sewage. ASVs that did not align with the FreshTrain database or custom human fecal and sewer databases were considered to come from different sources (e.g., terrestrial) and were classified as “uncategorized.”

### Quantitative PCR for human fecal marker.

To further examine relationships between microbial community parameters and potential human fecal contamination, a quantitative PCR (qPCR) assay for the human *Bacteroides* (HB) fecal marker was used (11, 12). Raw DNA extracts from each sample were used as the template for the qPCR, which followed previously published conditions (12). The concentration of HB marker in each sample was determined as the copy number (CN) per 100 ml of original water sample based on the volume of sample per filter and triplicate qPCR standard curves. Standard curves indicated an assay efficiency and *R*^2^ of 98.1% and 0.997, respectively. The limit of quantification was determined as the lowest quantifiable standard dilution (15 CN per reaction mixture volume), which is equivalent to 180 CN/100-ml sample. No-template controls were included for each qPCR run to ensure lack of contamination. As part of the laboratory’s quality assurance procedures, a subset of all samples processed in the laboratory during this time period were also tested for inhibition using a salmon sperm spike-in (16). No inhibition was detected.

### Statistical analysis.

Additional metadata for each sampling site, including watershed area, land use percentages, and stream order, were obtained from the USGS StreamStats and WiDNR 24K Hydro databases. Alpha diversity for each sample was calculated using both Shannon and Simpson indices in vegan 2.5-6 (34). Correlations between alpha diversity and categorical variables (historical water quality and dominant land use) were tested using Wilcoxon rank sum tests. Correlations between alpha diversity and continuous variables (percent developed land, water temperature, and dissolved oxygen) were tested using Pearson’s product-moment test for normally distributed variables or Spearman’s rank sum test for nonnormally distributed variables. To evaluate beta diversity, Bray-Curtis dissimilarity was calculated for the full data set and plotted using a nonmetric multidimensional scaling (NMDS) ordination. Depending on the hypotheses tested, Bray-Curtis dissimilarity was also calculated using subsets of data based on their taxonomic assignments in either freshwater or human sewage databases. PERMANOVA was used to test associations between Bray-Curtis dissimilarity and sample metadata (i.e., impacted versus good water quality and rural versus urban land uses).

Previous work using quantitative microbial risk assessment has determined that an HB concentration of 7,800 CN/100 ml indicates an excess illness risk of 0.03 (16). To determine the approximate relative abundance of sewage sequences that would be associated with this risk threshold, we evaluated the following equations using a Monte Carlo simulation:

$$f=\frac{C_{\mathrm{river,HB}}}{C_{\mathrm{sewage,HB}}}$$

$$r=\frac{C_{\text{sewage,cells}}\times f}{C_{\text{river,cells}}\times (1-f)}$$

where *f* is the volumetric fraction of sewage in the river, *C*_river,HB_ is the concentration of HB in the river, *C*_sewage,HB_ is the concentration of HB in sewage, *C*_sewage,cells_ is the total cell concentration in sewage, *C*_river,cells_ is the total cell concentration in the river, and *r* is the relative abundance of sewage sequences. These equations are based on an assumption that the relative abundance of an individual ASV generated using amplicon sequencing generally reflects the relative abundance of that sequence in the volume of sample analyzed. *C*_river,HB_ was set at the health risk threshold of 7,800 CN/100 ml. All other input variables (*C*_sewage,HB_, *C*_sewage,cells_, and *C*_river,cells_) were represented as log-normal distributions based on data collected previously by our laboratory and/or in published literature. Specific input data sources and distributions are described further in the supplemental material.

Samples were also divided categorically into risk (≥7,800 CN/100 ml), quantifiable (180 to 7,800 CN/100 ml), detectable (detected and <180 CN/100 ml), or undetectable (below limit of detection) HB contamination. Between these categories, indicspecies (70) was used with either sewer and stool or freshwater communities to determine the strength of these communities’ prediction for high HB abundance. Strongly associated ASVs were considered to be those with both sensitivity and specificity greater than 80%.

All data processing was performed in Linux command line, R version 4.0.0 with Rstudio version 1.2.5042, and Microsoft Excel.

### Data availability.

Raw sequence data are deposited in the NCBI Sequence Read Archive (BioProject PRJNA665728).
