# Supplementary material for: Human Fecal Contamination Corresponds to Changes in the Freshwater Bacterial Communities of a Large River Basin
Source: Microbiol Spectr. 2021 Sep 8;9(2):e01200-21. doi: 10.1128/Spectrum.01200-21 (PMC8557911; doi:10.1128/Spectrum.01200-21)
Supplement: SUPPLEMENTAL FILE 1 — Supplemental material. Download SPECTRUM01200-21_Supp_1_seq4.pdf, PDF file, 0.2 MB [file spectrum01200-21_supp_1_seq4.pdf]

Supplemental Material for

Human fecal contamination corresponds to changes in the freshwater and sewage bacterial communities of a large river basin

Jill S. McClary-Gutierrez[1], Zac Driscoll[2], Cheryl Nenn[2], Ryan J. Newton[1]

[1]School of Freshwater Sciences, University of Wisconsin-Milwaukee, 600 E. Greenfield Ave., Milwaukee, WI, 53204, USA

[2]Milwaukee Riverkeeper, 600 E. Greenfield Ave., Milwaukee, WI, 53204, USA

Materials & Methods

Sample site selection. A total of 16 sampling sites were selected for this study, including 4 sites in urban areas with good water quality, 4 sites in urban areas with impaired water quality, 4 sites in rural areas with good water quality, and 4 sites in rural areas with impaired water quality. Rural sites are located in the northern portion of the watershed and urban sites are located in the southern portion of the watershed. Impaired water quality status was based on atypical baseline dissolved oxygen (DO) monitoring data from 2005-2015. Sites with multiple baseline DO measurements below the state standard of 5 mg/L were designated as impaired. Additionally, one site (#10028907) had abnormally high DO measurements (minimum DO = 18.8 mg/L during 2015) and is known to receive large loads of fecal coliform (>5.6 billion CFU/year based on Total Maximum Daily Load modeling (1), and so this site was also categorized as impaired (Table S1).

Raw sewage sample data. To assign river sequences to human fecal and sewer sources, we used a 16S rRNA gene amplicon sequencing dataset previously generated in our lab from monthly time-series sampling of raw sewage at two wastewater treatment plants in Milwaukee, WI, USA over a five year period (2). Sequencing data from this study are available on NCBI SRA BioProject PRJNA597057, and the dataset consists of a total of 94 samples. ASVs from these samples were previously assigned to human body site sources (oral, skin, stool, or vaginal) by exact alignment against samples from the Human Microbiome Project (HMP) database. Any sequences from raw sewage samples that did not exactly align to HMP sequences were assigned to the "sewer" source group. Final ASVs binned to each human body site source or sewer source, along with the custom code used to determine exact alignments, are available on github at https://github.com/NewtonLabUWM/Sewage_TimeSeries.

Monte Carlo input data and distributions. In order to estimate the relative abundance of sewage sequences corresponding to a human health risk threshold, we constructed a Monte Carlo simulation based on the assumption that the relative abundance of a sequence generated from amplicon sequencing is proportional to that sequence's relative abundance in the sample analyzed. Specific equations used for the Monte Carlo simulation are

described in the main text. To perform the simulation, we determined distributions for the concentration of human *Bacteroides* (HB) in sewage ($C_{sewage,HB}$), the concentration of total bacterial cells in sewage ($C_{sewage,cells}$), and the concentration of total bacterial cells in a river ($C_{river,cells}$). A log-normal distribution for $C_{sewage,HB}$ has been previously described in (3) based in 98 sewage samples collected in the Milwaukee area; thus this distribution was used to generate values for $C_{sewage,HB}$. To determine appropriate distributions for $C_{sewage,cells}$ and $C_{river,cells}$, we performed a literature search and identified six previous papers describing total cell concentrations in raw sewage (4–9) and four previous papers describing total cell concentrations in rivers (10–13). Where possible, data points from individual samples were extracted from figures in the cited papers using PlotDigitizer v2.6.8 (http://plotdigitizer.sourceforge.net/). If this data was not presented or individual data points could not be discerned, we instead used mean values presented in figures or paper text. A summary of data extracted from each source is listed in Table S2. Using log_{10}-transformed concentration data, Lilliefors test for normality indicated a log-normal distribution was an appropriate assumption for both $C_{sewage,cells}$ and $C_{river,cells}$ data ($p > 0.05$). Final log-normal distribution parameters used to draw input variables in our Monte Carlo simulation are described in Table S3.

Table S1. Summary of sample site locations and watershed characteristics.

Site ID	Latitude	Longitude	Historical Water Quality	Land Use	Stream Order	Percent Developed Land	Watershed Area (sq. mi)
203093	43.50703	-88.20221	impacted	rural	4	5.44%	59.2
413814	42.97271	-87.940094	good	urban	3	99.96%	9.28
463206	43.233696	-87.98552	good	urban	2	63.69%	2.37
603121	43.590572	-88.05042	impacted	rural	2	4.86%	4.51
673269	43.2067	-88.142746	good	urban	3	24.00%	6.47
10008817	43.348984	-87.9656	good	rural	2	8.79%	7.95
10012524	43.439926	-88.02636	good	rural	2	3.16%	2.45
10012525	43.432713	-88.016014	good	rural	2	3.53%	1.81
10028773	43.26544	-87.93427	impacted	rural	4	22.79%	12.9
10028907	43.33829	-88.003296	impacted	rural	4	11.52%	117
10029078	43.53914	-88.28139	good	rural	2	4.43%	2.01
10029949	43.16242	-87.93145	impacted	urban	2	77.35%	3.28
10030491	43.686623	-88.03359	good	rural	1	4.80%	4.01
10031613	43.0505	-88.04671	good	urban	3	90.11%	18.4
10032540	43.12353	-88.043434	impacted	urban	3	47.79%	20.5
10033651	43.16278	-88.06054	impacted	urban	2	68.62%	2.8
10042138	42.99737	-87.92616	impacted	urban	3	98.60%	18.6

Table S2. Summary of literature data extracted for determining total cell concentration distributions in sewage and river water.

	**Reference**	**Method**	**Data Description**	**Number of data points**
sewage	[4]	flow cytometry	average concentration	1
	[5]	fluorescence microscopy	average concentration	3
	[6]	flow cytometry	individual samples	10
	[7]	estimation based on assumed 10 fg DNA / cell	individual samples	4
	[8]	assumption from previous uncited literature	assumption	1
	this study	fluorescence microscopy	individual sample	1
	[9]	16S rRNA copies by qPCR	average concentration	3
rivers	[10]	flow cytometry	average concentration	7
	[11]	flow cytometry	individual samples	23
	[12]	fluorescence microscopy	individual samples	24
	[13]	flow cytometry	duplicate samples	69

Table S3. Summary of distribution parameters for Monte Carlo simulation input variables.

Variable	Units	Distribution of log_{10}-transformed data		References
		Mean	Standard Deviation	
$C_{sewage,HB}$	CN/L	8.442	0.2403	[3]
$C_{sewage,cells}$	cells/L	12.10	0.8701	[4-9], this study
$C_{river,cells}$	cells/L	9.291	0.5336	[10-13]

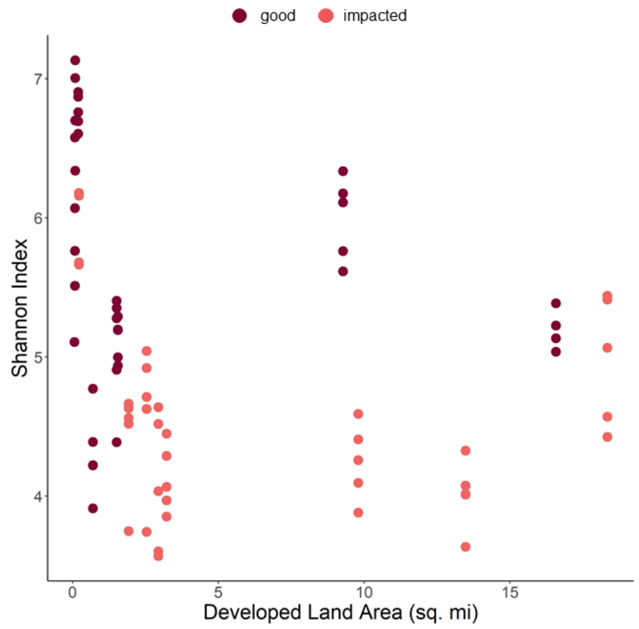

Figure S1. Association between developed land area in upstream watersheds and alpha diversity (Shannon index)

References

1. **CDM Smith**. 2018. Final Report: Total Maximum Daily Loads for Total Phosphorus, Total Suspended Solids, and Fecal Coliform, Milwaukee River Basin, Wisconsin.

2. **LaMartina E Lou**, **Mohaimani AA**, **Newton RJ**. 2021. Urban wastewater bacterial communities assemble into seasonal steady states. Microbiome **9**:1–13.

3. **McLellan SL**, **Sauer EP**, **Corsi SR**, **Bootsma MJ**, **Boehm AB**, **Spencer SK**, **Borchardt MA.** 2018. Sewage loading and microbial risk in urban waters of the Great Lakes. Elem Sci Anth **6**.

4. **Al-Jassim N**, **Ansari MI**, **Harb M**, **Hong PY**. 2015. Removal of bacterial contaminants and antibiotic resistance genes by conventional wastewater treatment processes in Saudi Arabia: Is the treated wastewater safe to reuse for agricultural irrigation? Water Res **73**:277–290.

5. **Joseph SM**, **Battaglia T**, **Maritz JM**, **Carlton JM**, **Blaser MJ**. 2019. Longitudinal Comparison of Bacterial Diversity and Antibiotic Resistance Genes in New York City Sewage. mSystems **4**:1–15.

6. **Ma L**, **Mao G**, **Liu J**, **Yu H**, **Gao G**, **Wang Y**. 2013. Rapid quantification of bacteria and viruses in influent, settled water, activated sludge and effluent from a wastewater treatment plant using flow cytometry. Water Sci Technol **68**:1763–1769.

7. **Muela A**, **Orruño M**, **Alonso ML**, **Pazos M**, **Arana I**, **Alonso RM**, **Jiménez RM**, **Garaizabal I**, **Maguregui MI**, **Barcina I**. 2011. Microbiological parameters as an additional tool to improve wastewater treatment plant monitoring. Ecol Indic **11**:431–437.

8. **Saunders AM**, **Albertsen M**, **Vollertsen J**, **Nielsen PH**. 2016. The activated sludge ecosystem contains a core community of abundant organisms. ISME J **10**:11–20.

9. **Timraz K**, **Xiong Y**, **Al Qarni H**, **Hong PY**. 2017. Removal of bacterial cells, antibiotic resistance genes and integrase genes by on-site hospital wastewater treatment plants: Surveillance of treated hospital effluent quality. Environ Sci Water Res Technol **3**:293–303.

10. **Freese HM**, **Karsten U**, **Schumann R**. 2006. Bacterial abundance, activity, and viability in the eutrophic River Warnow, northeast Germany. Microb Ecol **51**:117–127.

11. **Henson MW**, **Hanssen J**, **Spooner G**, **Fleming P**, **Pukonen M**, **Stahr F**, **Thrash JC**. 2018. Nutrient dynamics and stream order influence microbial community patterns along a 2914 kilometer transect of the Mississippi River. Limnol Oceanogr **63**:1837–1855.

12. **Read DS**, **Gweon HS**, **Bowes MJ**, **Newbold LK**, **Field D**, **Bailey MJ**, **Griffiths RI**. 2015. Catchment-scale biogeography of riverine bacterioplankton. ISME J **9**:516–526.

13. **Van Rossum T V.**, **Peabody MA**, **Uyaguari-Diaz MI**, **Cronin KI**, **Chan M**, **Slobodan JR**, **Nesbitt MJ**, **Suttle CA**, **Hsiao WWL**, **Tang PKC**, **Prystajecky NA**, **Brinkman FSL**. 2015. Year-long metagenomic study of river microbiomes across land use and water quality. Front Microbiol **6**:1–15.
